# Adult Personal Network Characteristics Associated with Early Life Adversity

**DOI:** 10.1093/geroni/igaa057.3391

**Published:** 2020-12-16

**Authors:** Janae Briggs, Stephanie Child

**Affiliations:** University of California, Berkeley, Berkeley, United States

## Abstract

Early life adversity (ELA) is associated with poor health through social and economic pathways. ELA also shapes cognitive and emotional development, including self-perception, social attachment and mental well-being. As such, ELA may shape later life health through social relationships, yet few studies have examined these associations. Data from the UC Berkeley Social Network Study were used to examine ELA measured retrospectively and current personal network characteristics among young (21-30 years) and older adults (50-70 years). ELA was operationalized as a summary of six experiences occurring before age 18 (e.g., parents’ divorce/separation, violence/drug use in the home, etc.). Personal network characteristics included objective measures, such as the number of ties who provide or receive various types of support, and subjective assessments about the adequacy of support received. Multivariate regression models adjusted for gender, race/ethnicity, and level of education. Among young adults, ELA was associated with more ties who rely upon the ego for support (b=0.15, 95% CI: 0.02, 0.28, p=0.02). Among older adults, ELA was associated with more ties named as either an advisor (b=0.14, 95% CI: 0.04, 0.21, p=0.02) or difficult/demanding (b=0.12, 95% CI: 0.04, 0.21, p<0.01). Furthermore, ELA was associated with less confidence in family support available (b= -0.09, 95% CI: -0.16, -0.03, p<0.01) and fewer emotionally close family members (b= -0.18, 95% CI: -0.32, -0.03, p=0.02) among older adults. In conclusion, clear differences emerged in network characteristics by exposure to ELA, particularly among older adults. The findings highlight potential pathways through which ELA patterns later life health.

